# Whole genome sequence data of *Chromobacterium violaceum* WCH4, a human pathogenic strain from Sabah, Malaysia

**DOI:** 10.1016/j.dib.2021.107533

**Published:** 2021-10-30

**Authors:** Vijay Kumar Subbiah, Zulina Mazlan, Nur Nashyiroh Mastor, Mohammad Zahirul Hoque

**Affiliations:** a Biotechnology Research Institute, Universiti Malaysia Sabah, Kota Kinabalu, Sabah, Malaysia; b Pathology Department, Sabah Woman & Children Hospital, Ministry of Health Malaysia, Kota Kinabalu, Sabah, Malaysia; c Department of Pathobiology & Medical Diagnostics, Faculty of Medicine and Health Sciences, University Malaysia Sabah, Kota Kinabalu, Sabah, Malaysia

**Keywords:** Chromobacterium violaceum, Genome sequence, Human pathogen, Antibiotic resistance

## Abstract

*Chromobacterium violaceum* is a gram-negative, facultative anaerobic bacillus which is commonly found in soil. It can cause mild diarrhoea upon infection but can progress, although rarely, to fatal multi-organ failure and death. Here we report the whole genome sequence data of *Chromobacterium violaceum* WCH4 strain, a pathogenic strain that was obtained from a 78 year old male patient suffering from an eye infection. This is a rare case of human infection of the bacteria. Blood culture report as well as 16S rRNA confirmed the presence of *C. violaceum* WCH4. DNA sequencing using the Illumina HiSeq 4000 system revealed a genome size of 4,637,406 bp with a GC-content of 64.89%. We identified 4,572 protein coding sequences (CDS), 78 transfer RNA (tRNA) genes, and 3 ribosomal RNA (rRNA) genes. The CDS included 1,261 hypothetical proteins and 3,311 proteins with functional assignments. We also identified seven putative genes involved in efflux pump and conferring multidrug antibiotic resistance. The genome data has been deposited at NCBI under the accession number JAFBBB000000000 and consist of full annotated genome and raw sequence data. Our data resource will assist in further downstream analysis and understanding of the mechanism of rare human infection caused by *Chromobacterium violaceum* WCH4 strain.

## Specifications Table

**Table:** 

Subject	Microbiology
Specific subject area	Genomics
Type of data	Raw Illumina's NGS paired-end sequencing reads in FASTQ format. Genome assembly data and assembled contigs in FASTA format. Data related to predicted genes and annotation of respective proteins.
How data were acquired	Whole genome was sequenced with an Illumina HiSeq 4000 system.
Data format	Raw and analyzed
Parameters for data collection	A specimen was obtained from an intravitreal tap of a 78 year old male patient. Initial blood culture report showed the growth of *C. violaceum* on MacConkey and blood agar, with its characteristic violet pigment. DNA was isolated from pure culture and DNA sequencing was performed to obtain whole genome data.
Description of data collection	Strain WCH4 was sequenced with the Illumina HiSeq 4000 platform using the Nextera XT DNA Sample Preparation Kit (Illumina, San Diego, CA). The library was then sequenced using Illumina's 2 × 150 bp sequencing chemistry.
Data source location	Eye Ward of the Women and Children Hospital, Likas, Kota Kinabalu, Sabah, Malaysian Borneo (6.01373 N 116.11938 E)
Data accessibility	This Whole Genome Shotgun project has been deposited at DDBJ/ENA/GenBank under the accession JAFBBB000000000 (www.ncbi.nlm.nih.gov/nuccore/ JAFBBB000000000). The version described in this paper is version JAFBBB010000000. All the details about the genome sequencing data are available on NCBI under BioProject accession number PRJNA698279 and can be accessed using the following link (https://www.ncbi.nlm.nih.gov/bioproject/ PRJNA698279).

## Value of the Data

• We present here the genome sequence data of *Chromobacterium violaceum* WCH4, which is a strain exhibiting rare human infection.
• The genome sequence data will be useful for medical researchers to perform comparative genomic studies of clinically and non-clinically strains of the bacteria.
• Genome data can be used to identify antibiotics resistance genes and to perform downstream cluster analyses to identify placement on the phylogenetic tree.
• We are concerned of recent cases of *C. violaceum* fatalities in Sabah. This work highlights that accurate bacterial identification and prompt treatment is essential to prevent serious consequences.

## 1 Data Description

*Chromobacterium violaceum* is a gram-negative, facultative anaerobic bacillus which is commonly found in soil and can cause mild diarrhoea upon infection but can progress, although rarely, to fatal multi-organ failure and death with some strains being antibiotic resistant [1], [2], [3], [4]. A 78 year old male patient was admitted to a public hospital in Kota Kinabalu, Sabah, for severe fever, diarrhoea with an eye infection. We isolated the pathogen from the intravitreal tap and confirmed its presence through 16S rRNA Sanger sequencing (Sequence given in Supplementary data-S1). The data was analysed using Blastn (https://blast.ncbi.nlm.nih.gov/Blast.cgi) with NCBI database and resulted with a positive hit to *Chromobacterium violaceum* with 99% query cover, 99.64% identity and 0.0 E value for the top 10 hits (Blast results are given in Supplementary data-S1). Subsequently, we performed whole genome sequencing of the pathogen. Here, we present the data on the whole genome sequence of the *C. violaceum* strain WCH4, which provides an initial glimpse of its pathogenicity. The data has been deposited to GenBank and can be viewed at www.ncbi.nlm.nih.gov/nuccore/ JAFBBB000000000 under the accession JAFBBB000000000 (Additional details are in the Specification Table).

In brief, a total of 5,061,114 paired reads of a 2 × 150-bp insert-size library using the NEBnext Ultra kit (New England Biolabs, NEB #E7645) were generated from the Illumina HiSeq 4000. These were then assembled into 37 contigs with a genome size of 4,637,406 bp at 325x coverage. The average G+C content was 64.89% and the N50 length, which is defined as the shortest sequence length at 50% of the genome was 434,055 bp (Table 1).

**Table 1:** Statistics of assembled sequence of *Chromobacterium violaceum* WCH4.

Assembly Statistics	Final Assembly
Number of pair-end reads	5,061,114
Sequencing Depth	325x
No of contigs	37
Genome length (bp)	4,637,406
Contig L50	5
Contig N50	434,055
GC%	64.89%
CDSs	4,572
tRNAs	78
Repeat regions	17
rRNA	3

**Fig. 1:**
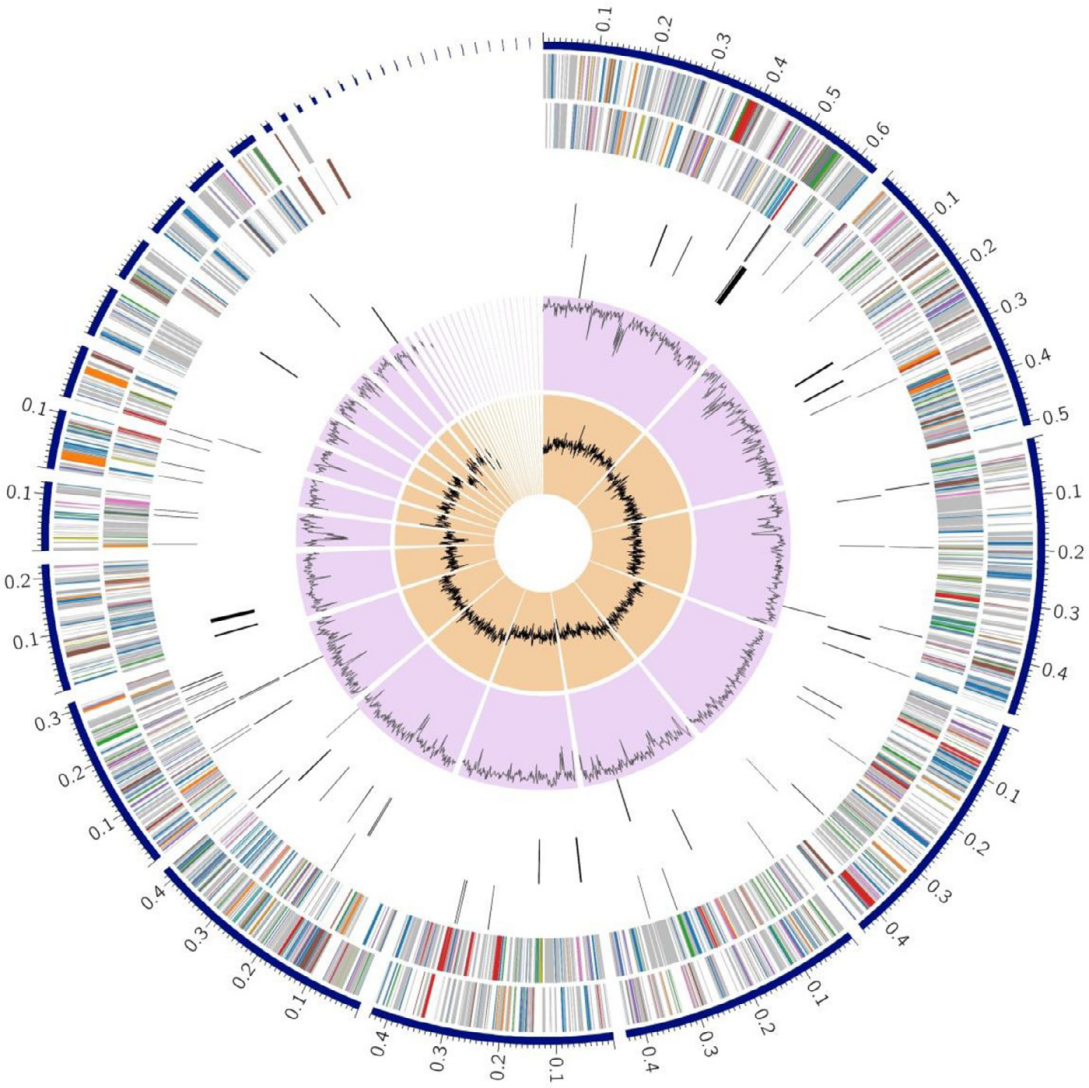
A circular graphical display of the distribution of the genome annotations of *Chromobacterium violaceum* WCH4. The concentric rings provide a snapshot view (from the outer most to the inner most rings) of a) the contigs, b) CDS on the forward strand, c) CDS on the reverse strand, d) RNA genes, e) CDS with homology to known antimicrobial resistance genes, f) CDS with homology to know virulence factors, g) GC content and h) GC skew. The 37 contigs (in Mbp) were arranged from the largest to the smallest in the outermost track. The numbers indicate the length of the contigs and only contigs larger than 100,000 bp are shown in the figure. The sizes of all contigs are given in supplementary data S2. The colours of the CDS can be mapped back to the subsystem categories given in Fig. 2.

This genome has 4,572 protein coding sequences (CDS), 78 transfer RNA (tRNA) genes, and 3 ribosomal RNA (rRNA) genes. The annotated genome contained 1,261 hypothetical proteins and 3,311 proteins with functional assignments (Table 2). Furthermore, annotated data on functional assignments included 994 proteins with Enzyme Commission (EC) numbers [5], 828 with Gene Ontology (GO) assignments [6], and 730 proteins that were mapped to KEGG pathways [7]. In addition, the PATRIC annotation includes two types of protein families [8], i.e. 4,272 proteins that belong to the genus-specific protein families (PLFams), and 4,318 proteins that belong to the cross-genus protein families (PGFams). A pictorial display of the genomic features of *Chromobacterium violaceum* WCH4 is given in the circular map of the genome in Fig. 1.

**Table 2:** Annotation of the *Chromobacterium violaceum* WCH4 with a breakdown of protein features.

Protein Features	Total
Hypothetical proteins	1,261
Proteins with functional assignments	3,311
Proteins with EC number assignments	994
Proteins with GO assignments	828
Proteins with Pathway assignments	730
Proteins with PATRIC genus-specific family (PLfam) assignments	4,272
Proteins with PATRIC cross-genus family (PGfam) assignments	4,318

**Fig. 2:**
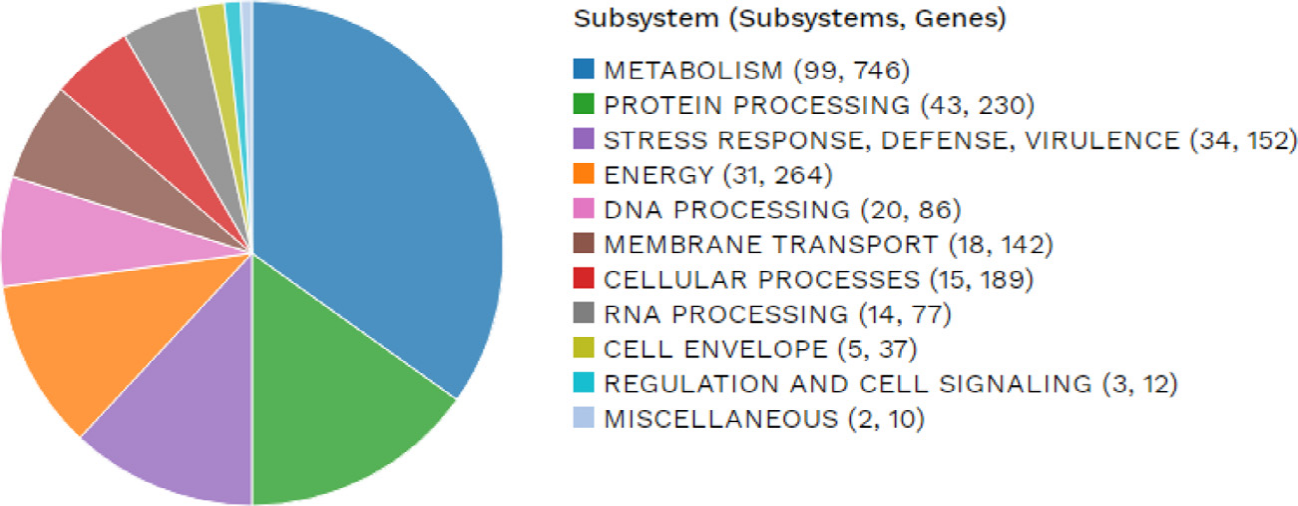
An overview of the subsystem categories assigned to the genes predicted in the genome of *Chromobacterium violaceum* WCH4. The whole-genome sequence of the strain WCH4 was annotated using the RAST server.

In addition, a subsystem analysis with the annotated genome was performed to determine the set of proteins that are part of a specific biological process or structural complex [9]. The RAST server-based annotation (using PATRIC) of the *C. violaceum* WCH4 genome resulted in a total of 284 subsystems representing 1,945 genes (Fig. 2). The distribution of the genes based on the subsystem category indicated that the top six highest genes were assigned to metabolism (746 genes), followed by energy production (264 genes), protein processing (230 genes), cellular processors (189 genes), stress response, defence and virulence (152 genes) and membrane transport (142 genes).

Furthermore, we provide a dataset on seven antimicrobial mechanisms and its corresponding 35 antimicrobial resistance (AMR) genes that was identified from the annotated genome (Table 3). These include antibiotic inactivation enzymes (2 genes), Antibiotic targets (20 genes), antibiotic target protection protein (1 gene), efflux antibiotic resistance (7 genes), genes conferring resistance via absence (1 gene), protein altering cell wall charge conferring antibiotic resistance (2 genes) and regulator modulating expression of antibiotic resistance genes (2 genes). The details of the RAST-based annotation are given in the supplementary data (S2). The genomic data reported here will pave the way for further study of the mechanism of pathogenicity of *Chromobacterium violaceum* WCH4.

**Table 3:** Data on antimicrobial resistance (AMR) mechanism and genes identified from the *Chromobacterium violaceum* WCH4 genome.

AMR Mechanism	Genes
Antibiotic inactivation enzyme	*AAC(6′)-Ic,f,g,h,j,k,l,r-z, ChpA family*
Antibiotic target in susceptible species	*Alr, Ddl, dxr, EF-G, EF-Tu, folA, Dfr, folP, gyrA, gyrB, inhA, fabI, Iso-tRNA, kasA, MurA, rho, rpoB, rpoC, S10p, S12p*
Antibiotic target protection protein	*BcrC*
Efflux pump conferring antibiotic resistance	*EmrAB-OMF, EmrAB-TolC, MacA, MacB, MdfA/Cmr, MdtABC-OMF, MdtABC-TolC*
Gene conferring resistance via absence	*gidB*
Protein altering cell wall charge conferring antibiotic resistance	*GdpD, PgsA*
Regulator modulating expression of antibiotic resistance genes	*EmrAB-TolC, OxyR*

## 2 Experimental Design, Materials and Methods

### 2.1 Bacterial strain isolation and identification

A specimen was obtained from an intravitreal tap of the patient. Initial blood culture report showed the growth of *C. violaceum* on MacConkey and blood agar, with its characteristic violet pigment. In addition, DNA sequencing of the 16S rRNA region on the ABI 3130 Genome Analyzer (Applied Biosystem, USA) confirmed the presence of *C. violaceum* strain WCH4. For whole genome sequencing, DNA was then isolated from pure bacterial culture using the conventional Phenol-Chloroform protocol [10].

### 2.2 Genome sequencing, assembly and annotation

The genomic DNA was converted into sequencing-ready library using the NEBnext Ultra kit (New England Biolabs, NEB #E7645). The library was then sequenced on the Illumina Hiseq 4000 (2 × 150-bp paired-end reads). We obtained approximately 5 million reads with a total of 1.5 Gb. The quality of the raw reads was analyzed by FastQC v0.11.9 software [11]. The sequences were then analysed at the Pathosystems Resource Integration Center (PATRIC) web server (https://www.patricbrc.org). The reads were was assembled using Unicycler v0.4.8 [12], an assembly pipeline for bacterial genomes at PATRIC. Filtering and polishing of the assembly was done using Pilon version 1.23 [13]. The genome was annotated using RAST tool kit (RASTtk) [14] through the PATRIC web server [15]. For functional assignments of proteins, we mapped proteins to Enzyme Commission (EC) [5], Gene Ontology (GO) [6], and KEGG pathways [7]. PATRIC annotation was used to assign genus-specific protein families (PLFams), cross-genus protein families (PGFams) [8] and subsystems [9]. In addition, a genome circular map was created using the ‘circular viewer’ functionality implemented in the PATRIC web server [15]. Classification of antimicrobial resistance (AMR) mechanism and genes was according to k-mer-based detection method, which utilizes PATRIC's curated collection of representative AMR gene sequence variants [15].

## Ethics Statement

This study was registered with the National Medical Research Register, Ministry of Health Malaysia (NMRR ID: No. 19-48-45702).

## CRediT Author Statement

**Vijay Kumar Subbiah:** Conceived and designed the experiments, Wet lab experiment,Data analysis and interpretation, Manuscript preparation, Contributed reagents/materials/analysis tools; **Zulina Mazlan:** Conceived and designed the experiments, Wet lab experiment, Manuscript preparation, Contributed reagents/materials/analysis tools; **Nur Nashyiroh Mastor:** Wet lab experiment, Data analysis and interpretation, Manuscript preparation, Contributed reagents/materials/analysis tools; **Mohammad Zahirul Hoque:** Conceived and designed the experiments, Data analysis and interpretation, Manuscript preparation. All authors read and approved the final manuscript.

## Declaration of Competing Interest

The authors declare that they have no known competing financial interests or personal relationships which have, or could be perceived to have, influenced the work reported in this article.

## Appendix Supplementary materials

Image, application 1

## References

[1] Yang C.H, Li Y.H. (2011). *Chromobacterium violaceum* infection: a clinical review of an important but neglected infection. J. Chin. Med. Assoc..

[2] da Gama A.M., de Almeida L.G., Yamane T., Spira B. (2018). Two draft genome sequences of *Chromobacterium violaceum* isolates from the Rio Negro. Genome Announc.

[3] Sneath P.H., Whelan J.P., Bhagwan Singh R., Edwards D. (1953). Fatal infection by *Chromobacterium violaceum*. Lancet.

[4] Donny Y., Jesse F.F.A., Azman Shah A.M., Simaa N.A., Tuba Thabitah A.T., Mariani R., Firdaus Ariff A.R.M., Rahmat T. (2018). Chromobacterium violaceum infection in two black-handed gibbons: a veterinary case report. Malaysian J. Vet. Res..

[5] Schomburg I., Chang A., Ebeling C., Gremse M., Heldt C., Huhn G., Schomburg D. (2004). BRENDA, the enzyme database: updates and major new developments. Nucleic Acids Res..

[6] Ashburner M., Ball C.A., Blake J.A., Botstein D., Butler H., Cherry J.M., Davis A.P., Dolinski K., Dwight S.S., Eppig J.T. (2000). Gene Ontology: tool for the unification of biology. Nat. Genet..

[7] Kanehisa M., Sato Y., Kawashima M., Furumichi M., Tanabe M. (2016). KEGG as a reference resource for gene and protein annotation. Nucleic Acids Res..

[8] Davis J.J., Gerdes S., Olsen G.J., Olson R., Pusch G.D., Shukla M., Vonstein V., Wattam A.R., Yoo H. (2016). PATtyFams: Protein Families for the Microbial Genomes in the PATRIC Database. Front. Microbiol..

[9] Overbeek R., Begley T., Butler R.M., Choudhuri J.V., Chuang H.Y., Cohoon M., de Crécy-Lagard V., Diaz N. (2005). The subsystems approach to genome annotation and its use in the project to annotate 1000 genomes. Nucleic Acids Res..

[10] Sambrook J., Fritsch E.F., Maniatis T. (1989).

[11] S. Andrews, Babraham bioinformatics-FastQC a quality control tool for high throughput sequence data, URL: https://www.bioinformatics.babraham.ac.uk/projects/fastqc, 2010.

[12] Wick R.R., Judd L.M., Gorrie C.L., Holt K.E. (2017). Unicycler: Resolving bacterial genome assemblies from short and long sequencing reads. PLoS Comput. Biol..

[13] Walker B.J., Abeel T., Shea T., Priest M., Abouelliel A., Sakthikumar S., Cuomo C.A., Zeng Q., Wortman J., Young S.K., Earl A.M. (2014). Pilon: an integrated tool for comprehensive microbial variant detection and genome assembly improvement. PLoS One.

[14] Brettin T., Davis J.J., Disz T., Edwards R.A., Gerdes S., Olsen G.J., Olson R., Overbeek R., Parrello B., Pusch G.D. (2015). RASTtk: a modular and extensible implementation of the RAST algorithm for building custom annotation pipelines and annotating batches of genomes. Sci. Rep..

[15] Wattam A.R., Davis J.J., Assaf R., Boisvert S., Brettin T., Bun C., Conrad N., Dietrich E.M., Disz T., Gabbard J.L. (2017). Improvements to PATRIC, the all-bacterial Bioinformatics Database and Analysis Resource Center. Nucleic Acids Res..

