# Supplementary material for: Whole genome sequence data of Chromobacterium violaceum WCH4, a human pathogenic strain from Sabah, Malaysia
Source: Data Brief. 2021 Oct 30;39:107533. doi: 10.1016/j.dib.2021.107533 (PMC8581251; doi:10.1016/j.dib.2021.107533)
Supplement: Supplementary file 1 [file mmc1.docx]

**Supplementary Data S1_16S rRNA Sequence of** ***Chromobacterium violaceum* WCH4 obtained from Sanger Sequencing on a ABI3130 Genome Analyser.**

16S rDNA PCR primers used were:

Forward (27F): 5’-AGAGTTTGATCMTGGCTCAG-3’

Reverse (1492R): 5’-GGTTACCTTGTTACGACTT-3’

>16S rRNA Sequence of Chromobacterium violaceum WCH4 (1406 bp)

AGTCgaacGGTAACAGGGtGCTtGCACCGCTGaCGaagtGGCGAACGGGtGagtAATGCGTCGGAATGTACCGTGTAATGGGGGaTAGCTCGGCGAAAGCCGGATTAATACCGCATACGCCCTGaGGGGGAAAGCGGGGGATCGAAAGACCTCGCGTTATACGAGCAGCCGACGTCTGATTAGCTAGTTGGTGAGGTAAGAGCTCACCAAGGCGACGATCAGTAGCGGGTCTGAGAGGATGATCCGCCACACTGGGACTGAGACACGGCCCAGACTCCTACGGGAGGCAGCAGTGGGGAATTTTGGACAATGGGGGCAACCCTGATCCAGCCATGCCGCGTGTCTGAAGAAGGCCTTCGGGTTGTAAAGGACTTTTGTCAGGGAGGAAATCCCGCTGGTTAATACCCGGCGGGGaTGACAGTACCTGAAgAATAAGCACCGGCTAACTACGTGCCAGCAGCCGCGGTAATACGTAgGGTGCGAGCGTTAATCGGAATTACTGGGCGTAAAGCGTGCGCAGGCGGTTGTGCAAGTCTGATGTGAAAGCCCCGGGcTTAACCTGGGAaCGGcATTGGAgACTGCACAGCTAGAGTGCGTCAgAGGGGGGtAGAAtTCCACGtgtAgcAgtGAAatGCgTAgagaTgtgGagGAaTAcCgaTgGcgaAGgcagCCCCctgGgaTgAcccTgacgctCaTGCacgAAAgcgtggGgagcaAAcaGgaTTAGAtaccCctGgtagtccacgCCcTAAAccGAtgttCaAcTAGCtgTtGGGGgTTTGAATCCttGGTAGcGTAGCTAACGCGTGAAGTTGaCCGcCtGGGGAGTAcggCCGCAAGGTTAAAACTCAAAGGAATTGaCGGGGACCCGCACAAGCGGTGGATGATGTGGATTAATTCGATGCAACGCGAAAAACCTTACCTGCTCTTGACATGTACGGAACTTGCCAGAGATGGCTTGGTGCCCGAAAGGGAGCCGTAACACAGGTGCTGCATGGCTGTCGTCAGCTCGTGTCGTGAGATGTTGGGTTAAGTCCCGCAACGAGCGCAACCCTTGTCATTAGTTGCCATCATTAAGTTGGGCACTCTAATGAGACTGCCGGTGACAAACCGGAGGAAGGTGGGGATGACGTCAAGTCCTCATGGCCCTTATGAGCAGGGCTTCACACGTCATACAATGGTCGGTACAGAGGGTTGCCAAGCCGCGAGGTGGAGCTAATCTCAGAAAACCGATCGTAGTCCGGATCGCACTCTGCAACTCGAGTGCGTGAAGTCGGAATCGCTAGTAATCGCAGATCAGCATGCTGCGGTGAATACGTTCCCGGGTCTTGTACaCaCCGCCCGTCACACCATGGGAGTGAGTTTCACCAGAAGTGGGTAGGCTAACCgCAAGGAGGCCGctaCcc

***Chromobacterium violaceum* WCH4 16S rRNA Blastn Sequences producing significant alignments**

| No. | Description | Query Cover | E Value | Percentage Identity | Genbank Accession Number |
| --- | --- | --- | --- | --- | --- |
| 1. | *Chromobacterium violaceum* strain 08022018 16S ribosomal RNA gene, partial sequence | 99% | 0.0 | 99.64% | MH790126 |
| 2. | *Chromobacterium violaceum* strain 726249W 16S ribosomal RNA gene, partial sequence | 99% | 0.0 | 99.64% | MG938493.1 |
| 3. | *Chromobacterium violaceum* strain 726249P 16S ribosomal RNA gene, partial sequence | 99% | 0.0 | 99.64% | MG938492.1 |
| 4. | *Chromobacterium violaceum* strain BF-R1 16S ribosomal RNA gene, partial sequence | 99% | 0.0 | 99.64% | KY292417.1 |
| 5. | *Chromobacterium violaceum* strain M-X1F 16S ribosomal RNA gene, partial sequence | 99% | 0.0 | 99.64% | KJ806351.1 |
| 6. | *Chromobacterium violaceum* strain FDAARGOS_1273 chromosome, complete genome | 99% | 0.0 | 99.64% | CP069587.1 |
| 7. | *Chromobacterium violaceum* strain FDAARGOS_1274 chromosome, complete genome | 99% | 0.0 | 99.64% | CP069442.1 |
| 8. | *Chromobacterium* sp. HL-VL 16S ribosomal RNA gene, partial sequence | 99% | 0.0 | 99.64% | JF734316.1 |
| 9. | *Chromobacterium violaceum* strain CV09 16S ribosomal RNA gene, partial sequence | 99% | 0.0 | 99.64% | FJ753567.1 |
| 10. | *Chromobacterium violaceum* strain HHM 1 16S ribosomal RNA gene, partial sequence | 99% | 0.0 | 99.64% | MN880157.1 |

*Note: The top 10 hits were all Chromobacterium violaceum with identical values for Query cover, E value and percentage identity.*
